# Pathophysiology and Treatment of Stroke: Present Status and Future Perspectives

**DOI:** 10.3390/ijms241914848

**Published:** 2023-10-03

**Authors:** Anna Bersano, Laura Gatti

**Affiliations:** Laboratory of Neurobiology and UCV, Neurology IX Unit, Fondazione IRCCS Istituto Neurologico Carlo Besta, 20133 Milan, Italy; laura.gatti@istituto-besta.it

Stroke is among the most prevalent causes of disability and is the second leading cause of death worldwide in Western countries [1]. It is a heterogeneous and complex disorder and may be ischemic (80% of cases) or hemorrhagic (about 20%) [1]. Conventional cerebrovascular risk factors, such as hypertension, hyperlipidemia, atrial fibrillation, diabetes, smoking and physical inactivity, are significant stroke risk factors. The major cause of stroke is embolism, either cardiac or caused by atherosclerotic plaques in the epiaortic vessels or aortic arch. In particular, strokes caused by atrial fibrillation are increasing and have been observed to be generally larger and more disabling than other kinds of strokes [2]. Intracranial atherosclerosis with in situ thrombosis and small vessel disease are also important mechanisms of stroke, whereas cervical artery dissection is one of the most prevalent causes of stroke in younger patients [3]. However, conventional risk factors are unable to explain all stroke cases, and the pathogenesis of stroke remains largely unknown. In fact, not all individuals with common cerebrovascular risk factors develop a stroke event, and stroke can occur in subjects without cerebrovascular risk factors [4]. Neuroinflammation has also been identified as a possible contributing pathophysiological mechanism, both in the acute and chronic phases. It can influence the response to damage, tissue repair and recovery, and post-stroke complications. The neuroinflammatory mechanism in stroke involves various cell types, including microglia, astrocytes, endothelial cells, and leukocytes, and the release of cytokines and chemokines (e.g., CXCL8, CCL2, and CCL3) and adhesion molecules [5]. Finally, epidemiological evidence supports the existence of a genetic susceptibility to common forms of stroke [6] and to mendelian disorders associated with stroke [7,8,9]. Therefore, the most common theory is that stroke represents a complex and multifactorial disease involving several lifestyles, genetic, environmental factors, as well as cellular and molecular signaling cascades that make the pathogenesis complex and the development of effective treatments extremely difficult. In fact, despite the enormous progress that has been reported in the last 5 years regarding the acute-phase therapy of stroke, including the continuous development of thrombolytic approaches and mechanical thrombectomy (MT) alone or in combination [10,11], most patients remain untreated due to the limited therapeutic windows and selection criteria. For a deeper understanding of the diverse pathological mechanisms and of the biological and molecular events that occur in stroke, and therefore for the development of novel or better tailored therapies, experimental animal models of ischemic stroke and in vitro cellular stroke models have been developed.

However, most of the existing animal models of cerebral ischemia or intracerebral hemorrhage are only imperfect representations of human stroke. Most stroke models are induced in healthy adult rodents instead of in elderly individuals with comorbidities, and the complex organization of the white matter is not comparable in rodent and human brains. Moreover, in most cases, best-practice guidelines for experimental models of stroke are not fully established, reflecting the failure of several neuroprotective and cellular therapies. 

For this Special Issue of *IJMS*, we encouraged the submission of review and original articles focusing on the most advanced and intriguing pathophysiological mechanisms of stroke and on pre-clinical experimental models that are highly relevant for the implementation of clinical studies.

Regarding possible pathophysiological mechanisms, with the advent of MT, occlusive clots have been made available for histological analysis, providing insights into the composition, structural organization and embolic origin [12]. 

In the present Special Issue, Essig et al., by analyzing 37 human thromboemboli obtained from acute ischemic stroke patients during MT, find that neutrophils are the main cellular component of cerebral thromboemboli. Neutrophils accumulate in the border region of fibrin-rich structures, thus indicating their possible interaction with distinct structural systems. They also find web-like NETs in 35 out of 37 thromboemboli, in varying amounts within fibrin-rich areas. They conclude that stroke etiology, age, and present oral anticoagulation are associated with morphological patterns and the number of neutrophils, providing insights into the knowledge of clot structural stability and thrombolytic resistance [13].

To explore the pathophysiology of cerebral amyloid angiopathy (CAA), which is a major cause of spontaneous intracerebral hemorrhage and an important contributor to cognitive decline in elderly patients, Gatti et al. present a review on the key pathophysiological mechanisms of disease and the experimental models that facilitate an understanding of disease drivers and potential therapeutic targets [14]. Several pathogenic mechanisms, including an unbalance between the production and clearance of amyloid beta (Aβ) protein, the role of the neurovascular unit, as well as ‘the prion hypothesis’, are explored. The role of genetic factors is also evaluated as a possible disease trigger, although none of these mechanisms completely explain the pathogenesis of the disease. In vivo and in vitro experimental models are reported, but the authors evidence that, considering the late onset of the disease, transgenic mouse models display a variable degree of CAA and present the limitation of a short life span. However, the advantage of minimal expenses and facile genetic manipulation could make these models promising in providing key findings regarding disease pathophysiology. Since animal models for CAA do not completely resemble human CAA, the authors conclude that the integration of experimental results and clinical data is mandatory in order to understand the pathophysiology of CAA and, therefore, develop effective therapeutic strategies.

Regarding possible therapeutic targets and neuroprotective strategies for stroke, Bhattacharya et al. present a review on soluble receptors, which are known to be intriguing concentration-dependent factors with a role in cytoprotection and neuroinflammation [15]. The authors particularly focus on the soluble cluster of differentiation 36 and 163, and soluble lipoprotein-related protein 1 (sCD36, sCD163, and sLRP1, respectively); they evaluate their possible role as therapeutic targets of stroke, as they regulate the bioavailability of the hemoglobin and heme after red blood cell lysis. They highlight the roles that these soluble receptors exert in inflammation and oxidative stress, and their possible pharmacotherapeutic potential in improving stroke outcomes, although further scientific investigation is required to establish their efficacy in diagnosis and therapy.

In their review, Chung Yang Yeh et al. review the available results of recent clinical trials for acute ischemic stroke treatment with both a thrombolytic agent and MT, and evaluate trials on neuroprotective agents, with particular attention paid to the role of voltage-gated potassium channel Kv2.1 for neuroprotection [16]. The authors conclude that there are promising neuroprotectant peptides both in preclinical development and with clinical efficacy in Phase III trials, as in the case of nerinetide, that encourage research in this field and exhibit promise regarding their application in clinical practice. 

Wan Leung et al. investigate the neuroprotective effects of Emodin, a traditional Chinese medicinal herb with antioxidative and protective properties in ischemia/reperfusion injury in MCAO rats and PC12 cells exposed to oxygen-glucose deprivation [17]. Emodin was observed to reduce infarct volume and cell death following focal cerebral ischemia injury, restore PC12 cell viability and reduce the production of reactive oxygen species (ROS) and glutamate release under conditions of ischemia/hypoxia. Therefore, Emodin has neuroprotective effects against ischemia/reperfusion injury, both in vitro and in vivo, possibly through activating the ERK-1/2 signaling pathway. An enhanced understanding of the mechanisms underlying the neuroprotective effects of Emodin is required in order to determine its role as a neuroprotective agent in stroke.

In another experimental model, distal middle cerebral occlusion (dMCAO) type 2 diabetes mellitus (T2DM) stroke mice and cultured human brain microvascular endothelial cells (HBMECs) subjected to hyperglycemic and inflammatory injury are studied by Jiang et al. [18]. This study aimed to determine whether recombinant fibroblast growth factor 21 (rFGF21) is beneficial in improving long-term neurological outcomes via post stroke blood–brain barrier (BBB) protection mechanism damage in T2DM mice, through peroxisome proliferator-activated receptor gamma (PPAR) activation in the cerebral microvascular endothelium. The authors demonstrate that all abnormal changes are significantly prevented by rFGF21 administration initiated at 6 h after stroke and that rFGF21 protects against acute BBB leakage after diabetic stroke, which is partially mediated by increasing PPAR DNA binding and the mRNA expression of BBB junctional complex proteins. Therefore, the authors hypothesize that rFGF21 is a promising candidate for treating diabetic stroke patients. 

Melia-Sorolla et al. present a review paper on the advantages and limitations of the utilization of pigs, which are large mammals with interesting brain characteristics and wide social acceptance, as a possible alternative animal model to rodents in the study of the pathophysiology of stroke [19]. Pigs, in fact, have human-like highly gyrencephalic brains, more sophisticated white matter connectivity, and have a subarachnoid space surrounding the brain that resembles that of humans, thus mimicking the clinical condition. Pig models may provide a unique opportunity to study structural brain damage via non-invasive imaging techniques, and to assess the role of specific molecules in the pathophysiology of stroke. The main limitation of utilizing swine brains to model human stroke is the presence of the ‘rete mirabile’, making difficult the endovascular access required to produce focal brain ischemia. The fact that pig plasminogen possesses resistance to tissue plasminogen activator (tPA)-mediated activation, and the significant contribution of both posterior and anterior cerebral arteries to the circulation via the circle of Willis, are additional limitations of the pig model. Therefore, since it could represent a promising experimental model, it should be explored further for possible clinical translation.

Lastly, Hatekayama present a review of cell therapy clinical trials and an update on the use of polarized cell therapies based on protective cell phenotypes, which are currently employed in pre-clinical studies, to facilitate functional recovery after post-reperfusion treatment in patients with ischemic stroke [20]. In particular, non-neuronal stem cells, such as bone-marrow-derived mesenchymal stem/stromal cells and mononuclear cells, are safe because they lack the tumorigenesis effect, do not induce rejection/allergy, and do not pose ethical issues. Several studies have focused on them as a cell source for cell therapy trials. However, the results are controversial and more focused clinical trials should be performed to reach a conclusion. Nevertheless, the application of polarized microglia or peripheral blood mononuclear cells could represent promising therapeutic strategies after stroke due to their pleiotropic action.

In conclusion, this Special Issue, entitled “Pathophysiology and Treatment of Stroke: Present Status and Future Perspective”, highlights a number of research directions with regard to understanding pathophysiological mechanisms and identifying therapeutic targets in order to reduce the significant burden of stroke.
